# Supplementary material for: Membranes: A Variety of Energy Landscapes for Many Transfer Opportunities
Source: Membranes (Basel). 2018 Feb 22;8(1):10. doi: 10.3390/membranes8010010 (PMC5872192; doi:10.3390/membranes8010010)
Supplement: Supplementary file 1 [file membranes-08-00010-s001.pdf]

Supplementary Information

Membranes : a variety of energy landscapes for many transfer opportunities

Patrice BACCHIN[a,*]

[a]*Laboratoire de Génie Chimique, Université de Toulouse, CNRS, INPT, UPS, Toulouse, France*
*corresponding author: patrice.bacchin@univ-tlse3.fr

S1 – Development and main assumptions in the model

The two-fluid formalism is based on the application of the momentum and the mass balances on the dispersed phase and on the fluid phase (Jackson, 1997). This formalism allows to account for the different transport phenomena (and then forces) acting on the "particles" (that can be solute or colloids) and on the fluid. In a recent paper (Bacchin, 2017), the presence of a membrane has been accounted for in this model through the solving of these equations on an energy landscape. The parameter describing the energy landscape is the interfacial pressure, $\Pi_i\,(x,y,z)$ representing the colloid/membrane interactions. If neglecting the gravity forces and if considering isotropic pressures, the momentum and the mass balances for the dispersed phase (dispersed colloids or solubilized molecules), the fluid phase (water molecules) and by addition for the mixture are:

Momentum balance
On the dispersed phase

$$+\ n\boldsymbol{F}_{drag} \qquad -\nabla\Pi_{cc\,th} - \nabla\Pi_{cc\,mc} - \phi\nabla\Pi_i = 0 \qquad \textit{(S1-1)}$$

On the fluid

$$-\frac{\eta_m \boldsymbol{u_m}}{k_p} - n\boldsymbol{F}_{drag} - \nabla p + \eta_m\nabla^2\boldsymbol{u}_m + \nabla\Pi_{cc\,th} = 0 \qquad \textit{(S1-2)}$$

On the mixture

$$-\frac{\eta_m \boldsymbol{u_m}}{k_p} - \nabla p + \eta_m\nabla^2\boldsymbol{u}_m - \nabla\Pi_{cc\,mc} - \phi\nabla\Pi_i = 0 \qquad \textit{(S1-3)}$$

Mass balance
On the dispersed phase

$$\frac{\partial\phi}{\partial t} = -\nabla\cdot(\phi\boldsymbol{u_c}) \qquad \textit{(S1-4)}$$

On the fluid

$$\frac{\partial(1-\phi)}{\partial t} = -\nabla\cdot\left((1-\phi)\boldsymbol{u_f}\right) \qquad \textit{(S1-5)}$$

On the mixture

$$0 = \nabla\cdot\boldsymbol{u_m} \qquad \textit{(S1-6)}$$

The different contributions in these equations can be dissipative or elastic in nature (Bacchin, 2017) :

The dissipative contributions are :

- the drag force, $\boldsymbol{F}_{drag} = \frac{\boldsymbol{u_m}-\boldsymbol{u_c}}{m(\phi)}$, represents the forces due to the friction induced by the relative velocity between the phases (**colloid/fluid** friction)
- the viscous dissipation, $-\eta_m \nabla^2 \boldsymbol{u}_m$**,** due to the viscosity mixture in the Eqs. 2 and 3 (**fluid/fluid** friction induced by the shear)
- the viscous dissipation in the fluid due to the interface that can be link to a porous media permeability, $-\frac{\eta_m}{k_p}\boldsymbol{u_m}$ in the Eqs. 2 and 3 (**fluid/membrane** friction)

The elastic storage (non dissipative contributions) are :

- the thermodynamic (reversible) colloid pressure gradient , $\nabla \Pi_{cc\,th}$, that corresponds to the water activity difference (**colloid/colloid** interaction)
- the mechanical (non-reversible) colloid pressure, $\nabla \Pi_{cc\,mc}$, due the shear and the associated shear induced diffusion (**colloid/shear** interaction) or due the compressibility of an arrested deposit if any (**aggregated colloid/fluid** interaction)
- the interfacial pressure, $\phi \nabla \Pi_i$, in the Eq. 1 and 3 (**colloid/membrane** interaction)
- the pressure drop, $-\nabla p$, representing the energy dissipated in the system (**fluid/fluid** interaction)

If considering a process where only the reversible part of the osmotic pressure is present, $\nabla \Pi_{cc\,mc} = 0$, (i.e. no effect of shear induced diffusion and no consolidated deposit on the membrane), the momentum balance can be written :

$$\frac{\phi}{V_p}\frac{\boldsymbol{u_m}-\boldsymbol{u_c}}{m(\phi)} - \nabla \Pi_{cc} - \phi \nabla \Pi_i = 0 \qquad \textit{(S1-7)}$$

$$\frac{\eta_m}{k_p}\boldsymbol{u_m} + \nabla p + \phi \nabla \Pi_i = 0 \qquad \textit{(S1-8)}$$

The first equation means that the velocity of the colloids (or more exactly the force needed to have a colloid velocity larger than the mixture velocity) is induced by the gradient of osmotic pressure and is modified by colloid/membrane interactions. The second equation indicates that the mixture velocity is induced by the gradient of fluid pressure and is modified by colloid/membrane interactions.

S2 – Energy map model and Boltzmann exclusion

In the absence of advection, $u_c=u_m=0$, the equation 6 can be simplified:

$$\frac{d\Pi_c(\phi)}{dx} - \phi \frac{d\Pi_i(x)}{dx} = 0 \qquad \textit{(S2-1)}$$

If considering the ideal case, where the osmotic pressure for colloids follows the Van't Hoff law, $\Pi_c = kT\frac{\phi}{V_p}$, the differential equation can be written :

$$\frac{d\phi}{\phi} = \frac{V_p}{kT} d\Pi_i(x) \qquad \textit{(S2-2)}$$

When this expression is integrated through the exclusion barrier where the interfacial pressure is varying from 0 to $\Pi_{ic\,max}$, the partition coefficient representing the volume fraction on the exclusion boundary in the membrane side over the volume fraction on the exclusion boundary in the bulk side can be written :

$$K = e^{-\frac{V_p \Pi_{ic\,max}}{kT}} = e^{-Ex} \qquad \textit{(S2-3)}$$

The exclusion number, Ex, is then directly linked to the partition coefficient, K, through the Boltzmann exclusion induced by the energy map.

S3 – Integration along the membrane composite layers

The concentration profiles can be determined through the integration of the eq. 2. When considering the continuity of the mass flux through the dimension perpendicular to the membrane, x, a constant mass flux has to be considered. This mass flux is also the one coming out through the membrane:

$$u_c\phi = u_m\phi_p \tag{S3-1}$$

The equation 2 can then be written:

$$u_m\phi_p = \boldsymbol{u}_m\phi - V_p m(\phi)\left(\frac{d\Pi_c(\phi)}{dx} - \phi\frac{d\Pi_i(x)}{dx}\right) \tag{S3-2}$$

The gradient in osmotic pressure is related to a diffusive mass flux if defining the diffusion coefficient with the Stokes-Einstein generalized law:

$$D = m(\phi)kT\frac{d\Pi_c}{d\phi} \tag{S3-3}$$

If considering that the ideal case where the osmotic pressure follows the Van't Hoff law, $\Pi_c = kT\frac{\phi}{V_p}$ and leads to a constant diffusion coefficient, the equation S3-2 can be written as a classical convection-advection balance with an additional mass flux due to the interaction with the membrane.

$$u_m\phi_p = \boldsymbol{u}_m\phi - D\frac{d\phi}{dx} + V_p m(\phi)\frac{d\Pi_i(x)}{dx}\phi \tag{S3-4}$$

The mass flux due to the interaction with the membrane (already introduced in model accounting for membrane-colloid interaction, (Bacchin et al., 2011, 1995)) can be written as an advective flow with a velocity, u_{ic}, due to the interface/colloid interaction :

$$u_{ic} = V_p m(\phi)\frac{d\Pi_i(x)}{dx} \tag{S3-5}$$

This last velocity is constant is considering the specific case where the interfacial pressure is varying linearly with x (Fig. 2). In these conditions, the equation 6 can be simplified to:

$$(u_m - u_{ic})\phi - D\frac{d\phi}{dx} = u_m\phi_p \tag{S3-6}$$

This equation can be integrated through all the layers representing the membrane in order to determine the volume fraction profile (subscript are defined in Fig.1) :

Polarisation layer

The integration of the transport equation, $u_m\phi - D\frac{d\phi}{dx} = u_m\phi_p$, can link the volume fractions at the extremities of the polarization layer to the Peclet number defined for the polarization layer, $Pe_{PL} = \frac{u_m\delta_{PL}}{D}$:

$$\frac{\phi_{mi} - \phi_p}{\phi_b - \phi_p} = e^{Pe_{PL}} \tag{S3-7}$$

This equation can also be rewritten to express the ratio of the volume fraction at the boundary to the transmission, $\frac{\phi_p}{\phi_0}$

$$\frac{\phi_{mi}}{\phi_b} = Tr + (1 - Tr)e^{Pe_{CP}} \tag{S3-8}$$

Inlet Exclusion layer

The integration of the transport equation, $(u_m - u_{ic})\phi - D\frac{d\phi}{dx} = u_m\phi_p$, can link the volume fractions at the extremities of the inlet exclusion layer to the Peclet number defined for the exclusion layer, $Pe_{Exi} = \frac{u_m\delta_{Exi}}{D}$:

$$\frac{\phi_{mi} - \frac{Pe_{EXi}}{Pe_{EXi} - Ex}\phi_p}{\phi_{ei} - \frac{Pe_{EXi}}{Pe_{EXi} - Ex}\phi_p} = e^{Pe_{EXi} - Ex} \tag{S3-9}$$

Membrane

The integration of the transport equation, $u_m\phi - D\frac{d\phi}{dx} = u_m\phi_p$, can link the volume fractions at the extremities of the membrane to the Peclet number defined for the membrane, $Pe_{MB} = \frac{u_m\delta_{MB}}{D}$:

$$\frac{\phi_{mo} - \phi_p}{\phi_{mi} - \phi_p} = e^{Pe_{MB}} \tag{S3-10}$$

Outlet exclusion layer

The integration of the transport equation, $(u_m - u_{ic})\phi - D\frac{d\phi}{dx} = u_m\phi_p$, can link the volume fractions at the extremities of the outlet exclusion layer to the Peclet number defined for the exclusion layer, $Pe_{Ex0} = \frac{u_m\delta_{Ex0}}{D}$:

$$\frac{\phi_{eo} - \frac{Pe_{EXo}}{Pe_{EXo} + Ex}\phi_p}{\phi_{mo} - \frac{Pe_{EXo}}{Pe_{EXo} + Ex}\phi_p} = e^{Pe_{EXo} + Ex} \tag{S3-11}$$

where $\phi_{eo}$is equal to the volume fraction of the permeate ϕ_p.

S4 - Expression of the transmission

The equations given in S3 can be combined in order to determine the solute transmission :

$$\frac{\phi_p}{\phi_0} = \frac{1}{1+Ex\left(\frac{(e^{-(Pe_{EXo}+Ex)}-1)e^{-Pe_{MB}-Pe\,cp-Pe_{EXi}+Ex}}{Pe_{EXo}+Ex}+\frac{\left(1-e^{-Pe_{EXi}+Ex}\right)e^{-Pe_{CP}}}{Pe_{EXi}-Ex}\right)} \quad \text{(S4-1)}$$

In the case of Péclet exclusion tending to 0 (for an infinitively thin membrane defined with a partition coefficient), the equation can be simplified:

$$\frac{\phi_p}{\phi_0} = \frac{1}{1+(e^{-(Ex)}-1)e^{-Pe_{MB}-Pe\,cp+Ex}-\left(1-e^{+Ex}\right)e^{-Pe\,cp}} \quad \text{(S4-2)}$$

$$\frac{\phi_p}{\phi_0} = \frac{1}{1+\frac{1-K}{K}\frac{1-e^{-Pe_{MB}}}{e^{Pe_{CP}}}} \quad \text{(S4-3)}$$

This last equation is the one given in (Bacchin et al., 1996; Opong and Zydney, 1991) for convective and diffusive transfer through membrane or for electrokinetic salt rejection (Jacazio et al., 1972).
If the polarization effect are negligible, $Pe_{CP} \rightarrow 0$:

$$\frac{\phi_p}{\phi_0} = \frac{1}{1+Ex\left(\frac{(e^{-(Pe\,exo+Ex)}-1)e^{-Pe\,m-Pe\,exi+Ex}}{Pe\,exo+Ex}+\frac{\left(1-e^{-Pe\,exi+Ex}\right)}{Pe\,exi-Ex}\right)} \quad \text{(S4-4)}$$

If the exclusion layers are infinitively thin $Pe_{Exi} and\ Pe_{Exo} \rightarrow 0$:

$$\frac{\phi_p}{\phi_0} = \frac{Ke^{Pe\,m}}{e^{Pe\,m}-(1-K)} \quad \text{(S4-5)}$$

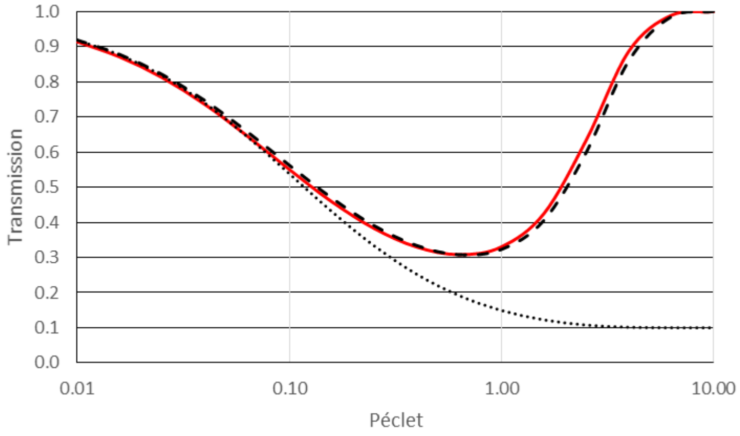

Fig. S1-1 : Variation of the transmission as a function of the Peclet number. The full line is the results of the full calculation with the following conditions $Pe_{EXo} = Pe_{EXi} = 0.1Pe_{MB} = 0.1Pe_{CP}$. The hyphen dashed line represent the calculation for the simplifying conditions of $Pe_{EXo}, Pe_{EXi} \rightarrow 0$. The dotted dashed line is the calculation when considering $Pe_{CP} \rightarrow 0$. For all calculations, the value of the partition coefficient is $e^{-Ex} = K = 0.1$.

S5 – Relationships for the counter pressure

From the model presented in the paper, the counter pressure can be expressed from different set of parameters. This section inventories the main relationships and gives the limits of these relationship for the conditions of thin exclusion layers.

Eq. 5 allows to determine the permeability of the membrane. When integrated along the layers of the membrane (Fig. 1), the counter pressure is expressed through the integration of the interfacial pressure, $\int \phi d\Pi_{ic}$. The gradient is only existing inside the exclusion layers. The integral can then be defined as $\int_{Exi+Exo} \phi d\Pi_{ic}$. The integral can be linked to the osmotic pressure difference and the drag force acting on the exclusion layer:

$$\int_{Exi} \phi d\Pi_{ic} = -\int_{Exi} d\Pi_c + \int_{Exi} \frac{\phi}{V_p} F_{drag} dx \quad (S5\text{-}1)$$

The filtration law can then be expressed as a function of the osmotic pressure difference and the drag forces inside the exclusion layers:

$$u_m = \frac{L_p}{\mu} \left(\Delta p_f - \Delta\Pi_{Exi} - \Delta\Pi_{Exo} - \int_{Exi+Exo} \frac{\phi}{V_p} F_{drag} dx \right) \quad (S5\text{-}2)$$

The drag force can be expressed as a function of the volume fraction inside the layers:

$$\int_{EXi} \frac{\phi}{V_p} \frac{u_m - u_p}{m} dz = \frac{u_m}{m_0 V_p} \int_{EXi} (\phi - \phi_p) dz \quad (S5\text{-}3)$$

The out-of-equilibrium counter pressure is then be expressed as the product of the drag force on immobile particles $\frac{u_m}{m_0}$ and the excess number of arrested colloids in the membrane $(\phi - \phi_p)/V_p$.

The concentration profile being determined (S3-9), this term can be defined for each layers according:

$$(u_m - u_{ic})\phi - D \frac{d\phi}{dz} = u_m \phi_p \quad (S5\text{-}4)$$

$$(u_m - u_{ic})(\phi - \phi_p) - u_{ic}\phi_p - D \frac{d\phi}{dz} = 0 \quad (S5\text{-}5)$$

$$(u_m - u_{ic}) \int_{EXi} (\phi - \phi_p) dz = D \int_{EXi} d\phi + u_{ic}\phi_p \delta \quad (S5\text{-}6)$$

$$\int_{Exi} (\phi - \phi_p) dz = \frac{D \int_{EXi} d\phi + u_{ic}\phi_p \delta}{(u_m - u_{ic})} \quad (S5\text{-}7)$$

$$\int_{Exi} (\phi - \phi_p) d\hat{z} = \frac{1}{Pe_{EXi} - Ex} \left(\phi_{mi} - \phi_{ei} + \phi_p Ex \right) \quad (S5\text{-}8)$$

The counter pressure can then be expressed as a function of the osmotic pressure at the boundary of the exclusion layers, $\Pi_{ei}, \Pi_{mi}, \Pi_{eo}, \Pi_{mo}$:

$$CP = \Pi_{ei} - \Pi_{eo} + \Pi_{mo} - \Pi_{mi} + \frac{Pe_{EXi}}{Pe_{EXi} - Ex} \left(\Pi_{mi} - \Pi_{ei} + \Pi_p Ex \right) + \frac{Pe_{EXo}}{Pe_{EXo} + Ex} \left(\Pi_{eo} - \Pi_{mo} - \Pi_p Ex \right) \quad (S5\text{-}9)$$

$$CP = \frac{Ex}{Ex - Pe_{exi}}(\Pi_{ei} - \Pi_{mi}) + \frac{Ex}{Ex + Pe_{exo}}(\Pi_{mo} - \Pi_{eo}) + \Pi_{eo}\frac{Ex^2(Pe_{exi} + Pe_{exo})}{(Pe_{exi} - Ex)(Pe_{exo} + Ex)} \quad \textit{(S5-10)}$$

The counter pressure can also be expressed as a function of the osmotic pressure at the extremities of the system membrane+ exclusion layers $\Pi_{ei}, \Pi_{mi}, \Pi_{eo}, \Pi_{mo}$ by considering the expression for the osmotic pressure at the vicinities of the membrane from concentration profile (S3-9 and S3-11) :

$$\phi_{mi} = \left(\phi_{ei} - \frac{Pe_{\,exi}}{Pe_{\,exi} - Ex}\phi_p\right)e^{Pe_{\,exi} - Ex} + \frac{Pe_{\,exi}}{Pe_{\,exi} - Ex}\phi_p) \quad \textit{(S5-11)}$$

$$\phi_{mo} = (\phi_{eo} - \frac{Pe_{\,exo}}{Pe_{\,exo} + Ex}\phi_p)e^{-Pe_{\,exo} - Ex} + \frac{Pe_{\,exo}}{Pe_{\,exo} + Ex}\phi_p \quad \textit{(S5-12)}$$

Then

$$CP = \frac{Ex}{Ex - Pe_{EXi}}\Pi_{ei}(1 - e^{Pe_{\,EXi} - Ex}) + \frac{Ex}{Ex + Pe_{exo}}\Pi_{eo}(e^{Pe_{\,EXi} - Ex} - 1) - \Pi_{eo}\frac{Ex}{Ex - Pe_{EXi}}\frac{Pe_{\,EXi}}{Pe_{\,EXi} - Ex}(1 - e^{Pe_{\,EXi} - Ex}) + \Pi_{eo}\frac{Ex}{Ex + Pe_{EXo}}\frac{Pe_{\,EXo}}{Pe_{\,EXo} + Ex}(1 - e^{-Pe_{\,EXo} - Ex}) + \Pi_{eo}\frac{Ex^2(Pe_{EXi} + Pe_{EXo})}{(Pe_{EXi} - Ex)(Pe_{EXo} + Ex)} \quad \textit{(S5-13)}$$

Or

$$CP = \Pi_{ei}(1 - e^{-Ex}) + \Pi_{eo}(e^{-Ex} - 1) + \frac{\Pi_{ei}(Exe^{-Ex}(1 - e^{Pe_{\,EXi}}) + Pe_{\,EXi}(1 - e^{-Ex})}{Ex - Pe_{EXi}} - \Pi_{eo}\frac{Ex}{Ex - Pe_{EXi}}\frac{Pe_{\,EXi}}{Pe_{\,EXi} - Ex}(1 - e^{Pe_{\,EXi} - Ex}) + \Pi_{eo}\frac{Ex}{Ex + Pe_{EXo}}\frac{Pe_{\,EXo}}{Pe_{\,EXo} + Ex}(1 - e^{-Pe_{\,EXo} - Ex}) + \Pi_{eo}\frac{Ex^2(Pe_{EXi} + Pe_{EXo})}{(Pe_{EXi} - Ex)(Pe_{EXo} + Ex)} \quad \textit{(S5-14)}$$

Similarly, the osmotic pressures Π_{ei} and Π_{eo} can be written as a function of the bulk osmotic pressure and the the permeate osmotic pressure by considering the concentration profile (S3-8 and S3-12)

$$\frac{CP}{\Pi_p Ex^2} = -\frac{(e^{-Pe_{EXi} + Ex} - 1)(e^{-Pe_{EXo} - Ex} - 1)e^{-Pe_{MB}}}{(Pe_{EXi} - Ex)(Pe_{EXo} + Ex)} + \left(\frac{1}{Pe_{EXi} - Ex}\right)^2(e^{-Pe_{EXi} + Ex} - 1) + \left(\frac{1}{Pe_{EXo} + Ex}\right)^2(e^{-Pe_{EXo} - Ex} - 1) + \frac{(Pe_{EXi} + Pe_{EXo})}{(Pe_{EXi} - Ex)(Pe_{EXo} + Ex)} \quad \textit{(S5-15)}$$

$$\frac{CP}{\Pi_b Ex^2} = \frac{\frac{Pe_{EXo} + Ex}{Pe_{EXi} - Ex}(e^{-Pe_{EXi} + Ex} - 1) + \frac{Pe_{EXi} - Ex}{Pe_{EXo} + Ex}(e^{-Pe_{EXo} - Ex} - 1) + Pe_{EXi} + Pe_{EXo} - (e^{-Pe_{EXi} + Ex} - 1)(e^{-Pe_{EXo} - Ex} - 1)e^{-Pe_{MB}}}{(Pe_{EXi} - Ex)(Pe_{EXo} + Ex) \text{ [illegible] } ((Pe_{EXi} - Ex) \text{ [illegible] } (Pe_{EXo} + Ex)(\text{[illegible]}) \text{[illegible]})}$$

(S5-16)

This last equation (that can also be written in a slightly different way accounting for the transmission as given in Eq. 6) allows to link the counter pressure to the operating conditions that are the different Péclet numbers and the exclusion number.

<u>Counter pressure limit for an infinitively thin membrane</u>

If considering an infinitively thin exclusion layer, $Pe_{EX} \rightarrow 0$, these equation are tending toward :

$$CP = \Pi_{ei}(1 - e^{-Ex}) + \Pi_{eo}(e^{-Ex} - 1) \quad (S5\text{-}17)$$

$$CP \to (\Pi_{ei} - \Pi_{eo})(1 - K) \quad (S5\text{-}18)$$

The counter pressure relationship can also be simplified when considering, $Pe_{EX} \to 0$

$$\frac{CP}{\Pi_b Ex^2} = \frac{-(e^{Ex}-1)-(e^{-Ex}-1)-(e^{+Ex}-1)(e^{-Ex}-1)e^{-Pe_{MB}}}{-Ex^2+Ex^2 e^{-Pe_{CP}}\left(\left(1-e^{Ex}\right)-(e^{-(Ex)}-1)e^{-Pe_{MB}+Ex}\right)} \quad (S5\text{-}19)$$

$$CP = -\Pi_b \frac{(1/K-1)+(K-1)+(1/K-1)(K-1)e^{-Pe_{MB}}}{(1-1/K)(1-e^{-Pe_{MB}})e^{-Pe_{CP}}-1} \quad (S5\text{-}20)$$

$$CP = \Pi_b \frac{(1-K)e^{Pe_{CP}}}{1+\frac{Ke^{Pe_{CP}}}{(1-K)(1-e^{-Pe_{MB}})}} = \Pi_b(1-K)e^{Pe_{CP}}(1-Tr) \quad (S5\text{-}21)$$

If considering an ideal case for the osmotic pressure, $Tr = \frac{\phi_p}{\phi_b} = \frac{\Pi_p}{\Pi_b}$, the counter pressure can be expressed as a function of the transmembrane osmotic pressure :

$$CP = \Pi_b(1-K)\left(\frac{\Pi_{ei}}{\Pi_b} - \frac{\Pi_p}{\Pi_b}\right) \quad (S5\text{-}22)$$

It can be noted that this last writing is equivalent to the relationship including a Staverman coefficient equal to (1-K). The model is then coherent with the model classically used. It allows to give a new explanation for the counter osmotic pressure and a new framework for the dynamic description of the osmosis flow.

S6 – Possibilities of separation with pulsed flow through asymmetric membranes

If the transmission through a membrane depends on the direction of the flow, the transmission may have different values for permeation fluxes (or Peclet numbers) having the same amplitude but different directions (positive or negative). The application of a pulsed flow through the membrane should make it possible to work with a series of filtration steps with a different transmission across the membrane as outlined in Figure S6-1.

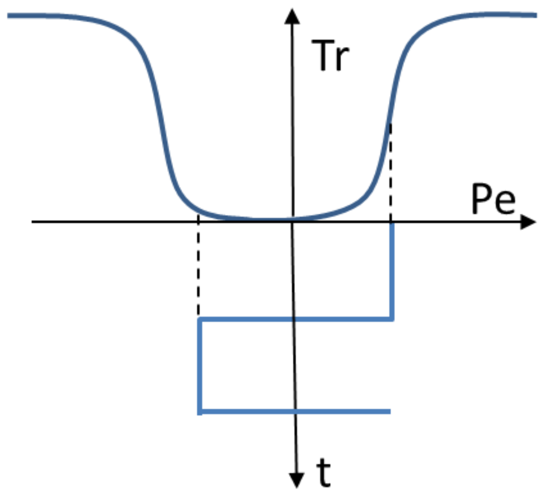

Fig. S6-1 : If the transmission is direction-dependent (top part of the figure), the application of pulsed flow with time (bottom part of the figure) should allow to have a difference in transmission for the filtration cycle realized in the different direction.

With this mode of filtration, a gradual increase in concentration in the compartment from which the transmission is lower should be expected. After a series of pulses, separation should then occur between the compartments without net overall filtration of the fluid through the membrane. It is interesting to note here that a pulsed flow (or shaking) could lead to a separation; when shaking and separation can be considered a priori as an antagonist.

Simple calculations taking into account a succession of stationary states can show how separation can occur. These calculations are based on the mass balances on the two compartments separated by the membrane. Fig. S6-2 shows the concentration in the left compartment and the right compartment during a flow pulsation. These calculations are performed for different conditions of asymmetry. When there is no asymmetry (left part in Fig. S6-2), the pulsed flow leads to the mixing and the concentration becomes identical. For weak asymmetry (determined from the experimental data presented in Fig. 7), the separation occurs after a series of square pulsed flows (median part of Fig. S6-2). This separation can be even faster in the case of a perfect diode with perfect transmission in one way and full retention in the opposite direction (right part in Figure S6-2).

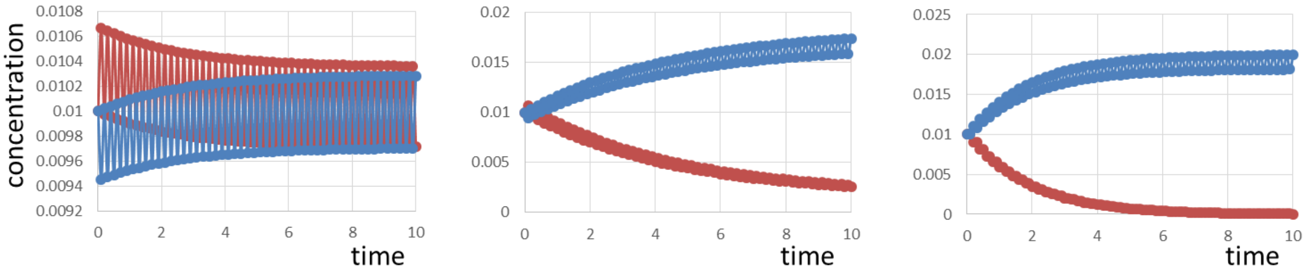

Fig. S6-2 : Concentration of the left compartment (in red) and the right compartment (blue) when filtrating with a square pulse flow through a membrane having a direction-dependent transmission. On the left, the transmission is symmetrical and equal to 0.4 for both directions; in the middle, transmission asymmetry is weak with a transmission of 0.4 from left to right and 0.1 from right to left; on the right, transmission asymmetry is maximum (perfect diode) with a transmission of 1 from left to right and 0 from right to left.

References

Bacchin, P., 2017. An energy map model for colloid transport. Chem. Eng. Sci. 158, 208–215. doi:10.1016/j.ces.2016.10.024

Bacchin, P., Aimar, P., Sanchez, V., 1996. Influence of surface interaction on transfer during colloid ultrafiltration. J. Membr. Sci. 115, 49–63. doi:10.1016/0376-7388(95)00279-0

Bacchin, P., Aimar, P., Sanchez, V., 1995. Model for colloidal fouling of membranes. AIChE J. 41, 368–376. doi:10.1002/aic.690410218

Bacchin, P., Marty, A., Duru, P., Meireles, M., Aimar, P., 2011. Colloidal surface interactions and membrane fouling: Investigations at pore scale. Adv. Colloid Interface Sci. 164, 2–11. doi:10.1016/j.cis.2010.10.005

Jacazio, G., Probstein, R.F., Sonin, A.A., Yung, D., 1972. Electrokinetic salt rejection in hyperfiltration through porous materials. Theory and experiment. J. Phys. Chem. 76, 4015–4023. doi:10.1021/j100670a023

Jackson, R., 1997. Locally averaged equations of motion for a mixture of identical spherical particles and a Newtonian fluid. Chem. Eng. Sci., Mathematical modelling of chemical and biochemical processes 52, 2457–2469. doi:10.1016/S0009-2509(97)00065-1

Opong, W.S., Zydney, A.L., 1991. Diffusive and convective protein transport through asymmetric membranes. AIChE J. 37, 1497–1510. doi:10.1002/aic.690371007
